# Supplementary figures and images for: Systems that support hearing families with deaf children: A scoping review
Source: PLoS One. 2023 Nov 27;18(11):e0288771. doi: 10.1371/journal.pone.0288771 (PMC10686551; doi:10.1371/journal.pone.0288771)

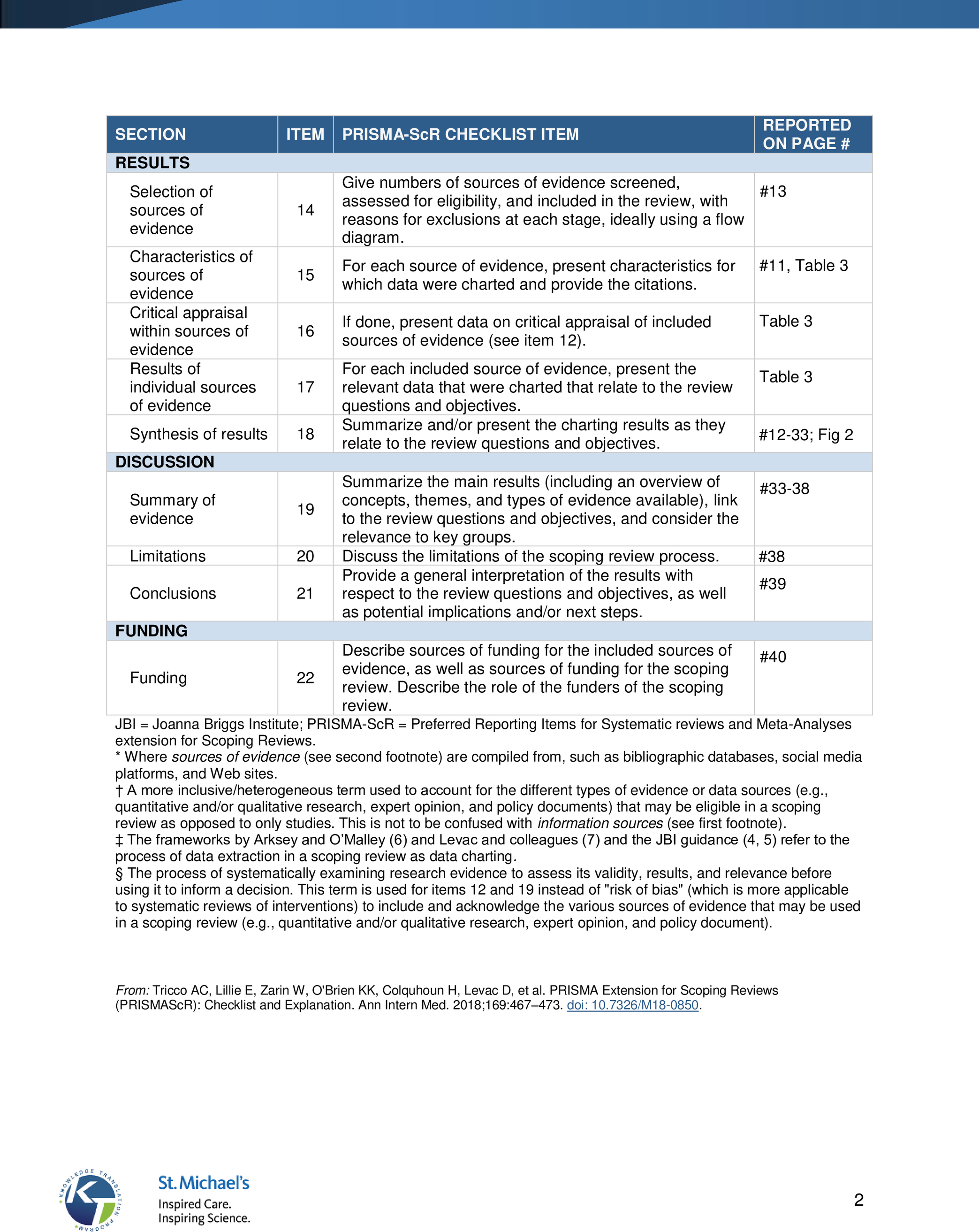

Supplement: S1 Checklist — (TIF) [file pone.0288771.s001.tif]
