# Supplementary material for: Systems that support hearing families with deaf children: A scoping review
Source: PLoS One. 2023 Nov 27;18(11):e0288771. doi: 10.1371/journal.pone.0288771 (PMC10686551; doi:10.1371/journal.pone.0288771)
Supplement: Attachment — Submitted filename: Response to reviewers PONE-22-29952.docx [file pone.0288771.s002.docx]

**Response to reviewers – PONE – 22 – 29952**

**Systems that support hearing families**

We are grateful to all reviewers for their helpful comments. Please see our responses below. On the tracked changes version of the manuscript, all new references in the References list are highlighted in yellow. The numbering both in the manuscript and in the reference list is now accurate for this revised manuscript.

**Reviewer #1:** Thank you for the opportunity to review Systems that support hearing families with deaf children: A scoping review. To help pinpoint the specific sentences that require my commentary in the absence of line numbers, I will use page numbers and the closest citation like this P:[X]

**Response**: Thank you, your comments across the paper are extremely helpful.

2:[4] - Support is not really defined here. I know it's not quite possible to define support as this is an individual issue, but the paper claims that support is lacking. What support would be needed?
**Response:** Thank you. A definition of support is now included in the first paragraph of the introduction.

3:[7,8] - Many people will find the term "developing countries" offensive. Perhaps Global South? Formerly colonized countries?
**Response** Thank you, now amended to ‘the Global South’.

3:[9] - List out these effects - perhaps cite Wyatte Hall's work on language deprivation syndrome.
**Response:** Thank you, effects and Wyatte Hall’s work now included.

3:[13] - This sentence a bit both-sides the debate. As you are not the New York Times, I don't think it's necessary to try to both-sides a clearly destructive argument from the Oralists. All deaf children should have some exposure to signed languages. Anything else is an enforcement of white supremacy values where only spoken languages are valued and everything else can be discarded.
**Response:** Helpful point, sentence with [13] now removed.

4:[17] - Let's be realistic. There is almost never signed alone. It is usually a mixture, or spoken languages only.
**Response:** Thank you, now rephrased.

5:[20] - Note that the width of the language development window is still under debate. 5 years isn't a hard stop. Language acquisition continues up until teenage years. But yeah. Early is good.
**Response:** Thank you and a fair point, now softened with ‘around the age of five years..’

5:[21] - This next paragraph is very good. I think you can add more citations to bolster your argument. Hall Hall and Caselli is good. I believe Lillo-Martin may have a recent paper.
**Response:** Very helpful, thank you, both suggested citations now added.

[22] - I don't think Geers et al is the right citation for this.
**Response:** Fair point, now removed.

6:[28] - I've seen several quasi definitions of support but nothing that makes me scream, yes, this is what support is. I would like to do this.
**Response:** Thank you, good point. We have provided a definition of support systems at this point.

7:[30] - Very nice and clear. I like the use of OSF
**Response:** Thanks.

8 - It's not immediately clear to me why educational databases were not searched as this would cover Journal of Deaf Studies and Deaf Education, Deaf Education & International, and the Annals of the Deaf
**Response:** Thanks. Whilst specific educational databases were not included as one of the four named databases, many of the included 65 papers are indeed from educational journals including Journal of Deaf Studies and Deaf Education, Deaf Education & International, and the Annals of the Deaf. The Proquest Central database does include educational databases. Also, due to the steering groups’ suggested search terms, the information specialist’s help and intense internet and hand searching for evidence the authors are confident that relevant evidence from educational journals is certainly included in this scoping review.

9 - I don't like the term hearing impaired but I wonder if using that term would net more articles.

**Response:** Agree, the term ‘hearing impaired’ was indeed used as a search term, please see Table 1, PICO framework.

10 - Does Rayyan software require a citation?
**Response:** Thanks, good point. Rayyan reference (Ouzzani et al now included).

11 - I think there's more funding to be honest. These are also Global North countries with robust economies. I am not sure this is as much an interest issue as it is an ability to receive funding.
**Response:** Thanks, sentence now revised.

11:[319 - I am not sure what reference 319 is, but I assume that will be fixed. However, I don't think that signed/spoken/both is the correct distribution of choices. It's usually both/spoken.
**Response:** Thanks, typo now corrected. The reference had been Yu (2021), but now changed to Hall, Hall and Caselli. Sentence now revised.

11: [19] - I am not sure that I agree that parents don't have time to engage with deaf communities (love the use of plural here). I think the answer is that there's usually not infrastructure to pipe parents to the appropriate deaf communities.
**Response:** Thanks, sentence now amended to reflect lack of infrastructure.

12:[18] - I want your point to be stronger here. Language does not interfere with language. Language does not hurt any kind of learning. Even asking the question is ableist.
**Response:** Thank you, sentence revised.

12:[35] - I'm surprised no one mentioned ableist attitudes towards signed languages.
**Response:** Thank you. Agree, ableist attitudes are not specifically stated as such in the literature, although that does appear to be what parent experience is describing.

13:[40] - Laura Mauldin's Made to Hear discusses some of this. Also mentions that parents tend to think that their kids will be the successful ones that don't need signed languages.
**Response:** Thank you, reference to Maudlin’s work now included.

15:[45] - I think this kind of hints that communication will always be a joint venture. I want you to say this stronger.

**Response:** Thank you, good point, sentence now revised.

16:[50] - These families really should have an option to learn the signed languages of their cultures (e.g. LSM)
**Response:** Thank you, a helpful addition and now included.

17:[52] - I think the issue of socio-emotional development being poor isn't well analyzed by hearing professionals. People react badly to not being able to communicate and things like Dinner Table Syndrome. Like, it's not the deafness. It's everything around it.
**Response:** Thank you, additional sentence now included.

21:[69] - I do not understand this sentence "Aware that by giving parents ‘the means to communicate with their deaf child by using the child’s primary language’ parents are provided with greater opportunity for understanding their child’s world. "
**Response:** Thank you, sentence now revised.

21:[69] - This paragraph is rough. I don't understand what the point is. It doesn't matter if the child overtakes the parent in proficiency. It only matters if the parents are the only model, like, forever.
**Response:** Thank you, paragraph now revised.

24:[77] - Without any context, reporting the data by race/ethnicity does not add positive information. Why do Black families struggle with coping strategies? How does the inherent racism of the US system restrict use of coping strategies? This information isn't here. I would remove everything after "Additionally,"
**Response:** Thank you, now revised and sentence removed.

25:[84] - Marschark is famous for never considering the context that the data is gathered in. Why are these kids feeling lonely? What does it mean that kids implanted earlier feel less lonely? Does anyone notice that the number of pirates decreased as the weather got warmer (https://pastafarians.org.au/pastafarianism/pirates-and-global-warming/)?
**Response:** Thanks, additional sentence added.

25:[85] - The weirdness of conflated variables appears in Holt et al's work. I see Kronenberger and Pisoni also contributed to this. This data result is silly. How would self reported control lead to small vocabularies? How would organization mean fewer inhibition problems? If a butterfly farts in the Atlantic, is there guaranteed to be a hurricane in the Pacific?

**Response:** Thank you for drawing attention to this. We have returned to the paper and revised the phrasing.

26:[86] - Sad that most of the deaf clubs in the states have closed.
**Response:** Thank you, sentence added and citations added.

27:[91] - I would not cite Johnston here. I think Kuster's work is more applicable. As is de Muelder.
**Response:** Thank you. We have retained reference to the Johnston paper, as we are referring to the retrieved paper from the included 65. We have supported this with a citation from Kusters, DeMeulder and O’Brien to increase clarity.

30:[101] - Make sure you're citing Leala Holcomb's work here.
**Response:** Thank you, we have now added a citation on Holcomb’s work.

31:[40] - Honestly it's fair to say the hearing parents are ableist and audist yeah.
**Response:** Thank you, point revised and made more clearly.

31:[61] - In my US State, all the parent supporters are oralist advocates. We have no signing parent mentor.

**Response:** Thank you, agree this may vary by region hence no changes made, as point is more about Deaf mentors, few Deaf professionals and supporting parents being a welcome intervention.

**Reviewer #2:**

Suggestion:
p. 5, sentence beginning ‘Sign Language often comes naturally to deaf children…’ — Rather than beginning the sentence with ‘Sign Language’ with both words capitalised, I recommend using lower case in ‘language’ because there is no language named ‘Sign Language’ but with both letters capitalized, it can create the impression that it is the name of a language. This reinforces the common misconception that there is only one sign language worldwide. By saying “sign language” with lowercase letters (or ‘signed languages’), it reads more parallel to ‘spoken language’ where no one would mistake that for a single language but rather a category of languages in a particular modality. This is done later in the MS (and even later in this paragraph) and it reads much more clearly without this possibility of misunderstanding.

**Response:** Thank you, very helpful, and now checked across the manuscript and only appears as ‘sign language or ‘signed language’, unless at the start of a sentence or in reference to a specific such as ASL or BSL.

p. 11, first sentence under subheading — perhaps a typo in the citation. It is listed as ‘[319’ with no closing bracket

**Response:** Thank you, it was a typo, now amended.

Throughout: It seems you mostly chose to capitalise ‘deaf’, which is fine, however this is a bit inconsistent, e.g., with some lowercase tokens on page 16. I’d suggest explaining whatever decision you make, citing the relevant literature and then being consistent throughout. If you’re not aware of this paper, I recommend it for a newer take on ‘Deaf’ versus ‘deaf’
<https://www.scirp.org/journal/paperinformation.aspx?paperid=97416>

**Response:** Thank you, we were not previously aware of this paper, which is extremely helpful and is now cited in our explanation. We have now included an Authors’ note at the start of the paper to provide reader clarity. Throughout the paper all reference to Deaf adults, communities, professionals, clubs, identity, culture, mentors and family members, then a capital D is used. Whilst when referring to deaf children, and deafness a lower-case d for deaf is used.

Positionality statement:
It would be helpful, if permitted by the journal, to include a positionality statement for the two authors so readers know what lived experiences/expertise they bring to this work. That would, to my mind, tie in with some of the narrative about the findings reported in this paper.

**Response:** Thank you, a useful point to consider. Positionality statements can be useful for readers. We thought it helpful to add more description about the project’s steering group in the Methods’ section.

Just in case it is helpful/applicable, please see Lieberman et al (2022)
https://sites.bu.edu/lavalab/files/2022/06/Lieberman-Mitchiner-Pontecorvo-2022.pdf
See also Oyserman and de Geus (2021)
<https://www.degruyter.com/document/doi/10.21832/9781800410756-011/html>

**Response:** Very helpful to have these references. We have included them towards the end of the paper, thank you for bringing them to our attention.

**Reviewer #3:**

This paper is well-balanced and captures the nuance quite well of what parents experience when they learn of having a deaf child and how the medical model influences choices. I was impressed with the acknowledgement of how marginalized signed languages have been in deaf child development. I applaud the authors for bringing this balanced view and highlighting the plurality of options in an international scope. I have no major feedback on this paper, there are minor grammar and writing aspects that can be improved with editor review.

**Response -**Thank you for your comments.
