# Supplementary material for: Systems that support hearing families with deaf children: A scoping review
Source: PLoS One. 2023 Nov 27;18(11):e0288771. doi: 10.1371/journal.pone.0288771 (PMC10686551; doi:10.1371/journal.pone.0288771)
Supplement: Attachment — Submitted filename: Response to reviewers PONE-D-22-29952R1.docx [file pone.0288771.s003.docx]

PONE-D-22-29952R1

We are grateful to all reviewers for their helpful comments. Please see our responses below, and on the tracked changes version of the manuscript:

**Editor comments:**

1. Please re-word to use low-middle income countries and high-income countries as appropriate throughout the manuscript.

**Response**: Thank you for this helpful comment. Now changed throughout.

**Reviewer #2:** Overall, I’m very happy with the revisions to the manuscript. Thank you for your hard work in addressing reviewer comments. I have just one more remark:

1. P. 53-54 (paragraph straddling these pages). Here you state that an alternative may have been for these families to learn LSM, however, I’m looking at the original article, and I’m only seeing that the families in this study were from “Spanish-speaking” backgrounds. It does not specify their country (or territory, e.g., Puerto Rico) of origin. I do not feel the suggestion to specifically name LSM here is appropriate for two reasons. First, if the family is from, say, El Salvador, why would they be compelled to learn LSM? Second, if the family lives in the US and there is no one in their community who knows LSM, how is it functional for them to learn a language they can’t use with anyone? I feel strongly the manuscript would be better off with the sentence in question omitted.
I’m not sure if this would be helpful, but if you feel compelled to comment on some of the unique struggles of families from a Spanish-speaking background, you may consider this article:

Steinberg, A., Bain, L., Li, Y., Delgado, G., & Ruperto, V. (2003). Decisions Hispanic Families Make After the Identification of Deafness. Journal of Deaf Studies and Deaf Education, 8(3), 291–314. <https://doi.org/10.1093/deafed/eng016>

**Response**: Thank you for this helpful comment. On consideration we have removed this sentence.
