# Supplementary material for: Systems that support hearing families with deaf children: A scoping review
Source: PLoS One. 2023 Nov 27;18(11):e0288771. doi: 10.1371/journal.pone.0288771 (PMC10686551; doi:10.1371/journal.pone.0288771)
Supplement: Attachment — Submitted filename: Response to Reviewers_PONE-D-22-29952R2 .docx [file pone.0288771.s004.docx]

**Response to Reviewers- - PONE-D-22-29952R2**

Thank you for your helpful comments and guidance. As we have revised the manuscript, we have looked extensively at other scoping reviews published in Plos One, which has been helpful.

Reviewer comment: Please check your prisma flow diagram in figure 1and explain exclusion criteria inside the box. There is a need of improvement in PRISMA FLOW fig 1. Please see page 13. Justify exclusion screening (n=821). The diagram have made poorly. The line are irregular. Kindly redraw and elaborate in detail all criteria. Please do include it in text if any new addition in text.

Response: Thank you for your suggestions. The PRISMA diagram has been re-done. No further text added to manuscript.

Reviewer comment: Table 2, Strength and weakness must possess under a separate column. Rewrite in and elaborate the content.

Response: Separate columns have now been added for strengths and another for weaknesses, with additional content now added. A few entries do just state ‘small sample’, and we have been back to these articles again, but there is little else to say with regards to these publications due to limited detail.

Reviewer comment: I could not find the search strategy as additional file. Kindly check your manuscript. It is suggested to provide inside the manuscript.

Response: Apologies. An example of a search strategy from one database (CINAHL) is now included within the manuscript and has also been uploaded as an additional file.

Reviewer comment: Please write your manuscript according to Plosone criteria and provide following clear methodology in method section such as Data screening, data entry and collection method.

Response: Thank you. Further detail about data screening, data entry and collection method is now included on page 12. The sub-headings used in the Methods sections are based on Arksey and O’Malley’s framework, as explained on page 8.
